# A novel HDAC inhibitor, CG200745, inhibits pancreatic cancer cell growth and overcomes gemcitabine resistance

**DOI:** 10.1038/srep41615

**Published:** 2017-01-30

**Authors:** Hee Seung Lee, Soo Been Park, Sun A Kim, Sool Ki Kwon, Hyunju Cha, Do Young Lee, Seonggu Ro, Joong Myung Cho, Si Young Song

**Affiliations:** 1 Division of Gastroenterology, Department of Internal Medicine, Yonsei University College of Medicine, Seoul, Korea; 2 CrystalGenomics, Inc., 5F, Bldg A, Korea Bio Park, Seongnam, Korea

## Abstract

Pancreatic cancer is predominantly lethal, and is primarily treated using gemcitabine, with increasing resistance. Therefore, novel agents that increase tumor sensitivity to gemcitabine are needed. Histone deacetylase (HDAC) inhibitors are emerging therapeutic agents, since HDAC plays an important role in cancer initiation and progression. We evaluated the antitumor effect of a novel HDAC inhibitor, CG200745, combined with gemcitabine/erlotinib on pancreatic cancer cells and gemcitabine-resistant pancreatic cancer cells. Three pancreatic cancer-cell lines were used to evaluate the antitumor effect of CG200745 combined with gemcitabine/erlotinib. CG200745 induced the expression of apoptotic proteins (PARP and caspase-3) and increased the levels of acetylated histone H3. CG200745 with gemcitabine/erlotinib showed significant growth inhibition and synergistic antitumor effects *in vitro. In vivo*, gemcitabine/erlotinib and CG200745 reduced tumor size up to 50%. CG200745 enhanced the sensitivity of gemcitabine-resistant pancreatic cancer cells to gemcitabine, and decreased the level of ATP-binding cassette-transporter genes, especially multidrug resistance protein 3 (MRP3) and MRP4. The novel HDAC inhibitor, CG200745, with gemcitabine/erlotinib had a synergistic anti-tumor effect on pancreatic cancer cells. CG200745 significantly improved pancreatic cancer sensitivity to gemcitabine, with a prominent antitumor effect on gemcitabine-resistant pancreatic cancer cells. Therefore, improved clinical outcome is expected in the future.

Pancreatic cancer is the most lethal cancer with poor prognosis. Patients with locally advanced disease have a median survival time of 8–12 months, and patients with distant metastases have significantly worse outcomes, with a median survival time of only 3–6 months^1^. At present, gemcitabine-based regimens are considered standard treatment for pancreatic cancer patients. Recently, gemcitabine plus nab-paclitaxel showed minimal improvements in overall survival with a median survival approaching one year in patients with advanced pancreatic cancer^2^. Despite these advances in gemcitabine-based treatment, the number of pancreatic cancer-related deaths and chemotherapy refractory pancreatic cancer has continued to increase^3^. Since the resistance of pancreatic cancer negatively affects the therapeutic effects of gemcitabine, agents that increase tumor sensitivity and overcome drug resistance to gemcitabine are needed.

Histone deacetylase (HDAC) inhibitors are the most developed anti-cancer drugs targeting epigenetic modulation. In normal cells, histone acetylation is precisely controlled by histone acetyl transferase (HAT) and HDAC. However, hyper-acetylation of oncogenes or hypo-acetylation of tumor suppressor genes is frequently observed in cancer cells^4^. HDAC inhibitors acetylate the lysine residues at the histone N-terminal tail, and loosen the association of histones with DNA, thereby inducing the expression of genes related to tumor suppression and differentiation. Therefore, HDAC inhibition offers potential as an effective cancer treatment^5^,^6^. To date, two HDAC inhibitors, vorinostat and romidepsin, have been approved by the Food and Drug Administration (FDA) for the treatment of cutaneous T-cell lymphoma. Regarding pancreatic cancer, previous studies showed that HDAC inhibitors show *in vitro* and *in vivo* anti-tumor effects^7^,^8^,^9^,^10^.

CG200745, (E)-N(1)-(3-(dimethylamino)propyl)-N(8)-hydroxy-2-((naphthalene-1-loxy) methyl)oct-2-enediamide, is a recently developed HDAC inhibitor^4^,^11^,^12^. Similar to other inhibitors, such as vorinostat and belinostat, the novel HDAC inhibitor, CG200745, is an intravenous hydroxamate-based pan-HDAC inhibitor. Its inhibitory effect on cell growth has been demonstrated in several types of cancer cells, including prostate cancer, renal cell carcinoma, and colon cancer in mono- and combinational-therapy with other anticancer drugs^4^,^11^,^12^,^13^. CG200745 was five times more effective than vorinostat in acetylating histone H3 in colon cancer-cell lines, and induced the acetylation of the tumor suppressor, p53, and cancer cell death^11^.

Combination therapy using gemcitabine/erlotinib is an approved standard chemotherapy in patients with advanced pancreatic cancer, but has marginal therapeutic benefits^14^. To improve the therapeutic results, we investigated the anti-tumor effect of CG200745 combined with gemcitabine/erlotinib in pancreatic cancer cells. We also evaluated whether CG200745 could overcome the resistance to gemcitabine in human gemcitabine-resistant pancreatic cancer cells.

## Results

### Effect of CG200745 on growth inhibition and cell death in pancreatic cancer cells

**Figure 1:**
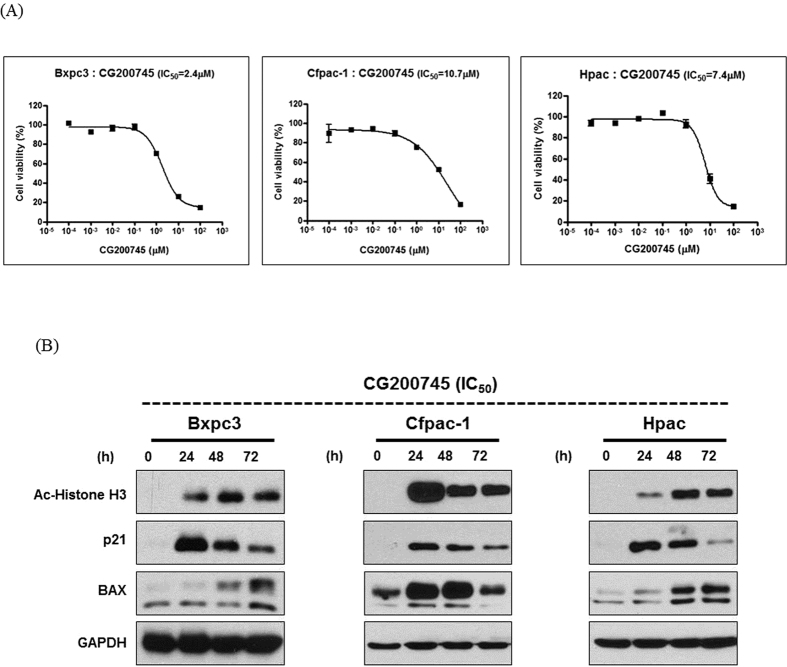
Anti-proliferative and pro-apoptotic activities of CG200745 against pancreatic cancer cells. (**A**) Cell viability curve based on the CG200745 concentration in three pancreatic cancer cell lines. CG200745 inhibits the proliferation of pancreatic cancer cells. (**B**) CG200745 induces histone-H3 acetylation and increases BAX and p21 expression related to apoptosis.

As shown in Fig. 1A, CG200745 dose-dependently decreased pancreatic cancer cell viability. To determine the inhibitory effects of CG200745 on cell proliferation, we measured the IC_50_ of CG200745 in pancreatic cancer cells. BxPC3 were more sensitive to the growth-inhibitory effect of CG200745 (IC_50_; 2.4 μM) than Cfpac-1 and HPAC (IC_50_; 10.7 and 7.4 μM, respectively). To assess the effects of CG200745 on HDACs in pancreatic cancer cells, we measured histone H3 acetylation levels. Treatment of pancreatic cancer cells with the IC_50_ of CG200745 caused a significant increase in histone H3 acetylation within 24 h of treatment (Fig. 1B). Doses of erlotinib and CG200745 equivalent to IC 20~30 were selected to minimize individual cytotoxic effect and know the combinatory anticancer effect on the pancreatic cancer cell lines, respectively (Fig. 1 and Supplementary Fig. 1). The effect of CG200745 on pancreatic cancer cell apoptosis was also tested. Western blot analysis indicated that CG200745 increased the expression of pro-apoptotic proteins, BAX, and p21 (Fig. 1B).

### Synergistic inhibitory and apoptotic effect of CG200745 combined with gemcitabine/erlotinib in pancreatic cancer cells

**Figure 2:**
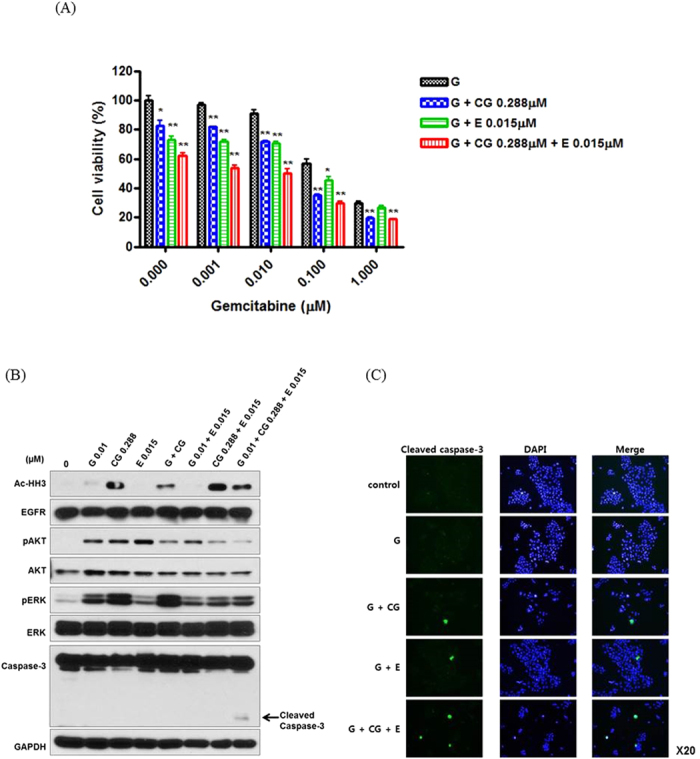
Synergistic effect of CG200745 combined with gemcitabine/erlotinib in pancreatic cancer cell lines (BxPC3). The doses of erlotinib and CG200745 were equivalent to IC 20~30. (**A**) The growth of pancreatic cells was analyzed via an MTT assay after treatment with various concentrations of gemcitabine over a time-course (0–72 h). The anti-proliferative effect of CG200745 with gemcitabine/erlotinib is more enhanced than the effect of gemcitabine/erlotinib without CG200745 in pancreatic cancer cells. (**B**) Western Blot analysis to investigate the pancreatic cancer cell apoptosis and analyze the molecular pathway related to CG200754. CG200745 combined with gemcitabine/erlotinib induces apoptosis through caspase-3 activation. (**C**) Immunofluorescent staining of cleaved caspase-3 expressing cells. Fluorescence signals specific to cleaved caspase-3 antibodies were visualized as green, and DAPI (blue) was used to indicate nuclei. * or **Indicates significant differences compared with the control (*p* < 0.05 or *p* < 0.01). G, gemcitabine; CG, CG200745; E, erlotinib.

BxPC3, Cfpac-1, and HPAC cell lines were treated with gemcitabine, erlotinib, and CG200745. The results from the cell viability indicated that the anti-proliferative effect of CG200745 with gemcitabine/erlotinib was significantly higher than that of other combinations (Fig. 2 and Supplementary Fig. 2). Western blot analysis showed the apoptotic protein, cleaved caspase-3, in a triple combination line. A low CG200745 concentration, with a combination of gemcitabine or erlotinib, significantly increased the antitumor effect, and was most effective when combined with both regimens.

**Figure 3:**
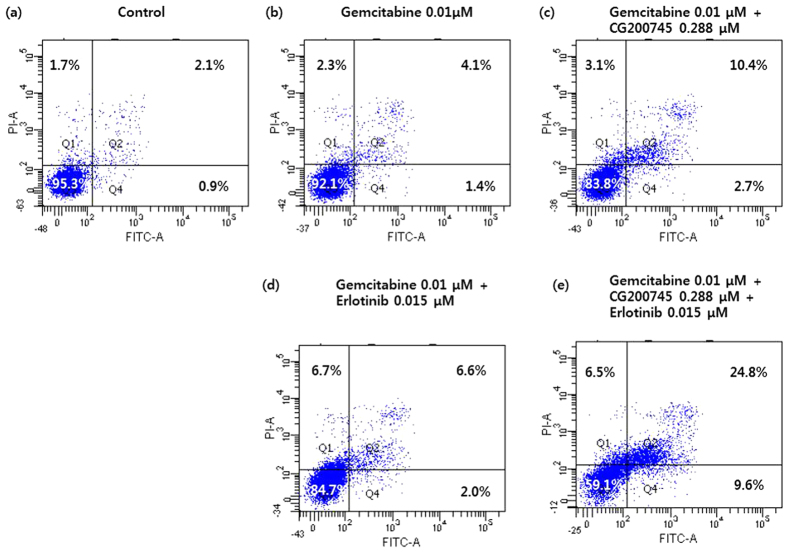
The FACS analysis indicates that CG200745 induces apoptosis of pancreatic cancer-cell lines. The doses of erlotinib and CG200745 were equivalent to IC 20~30. The apoptosis rate of BxPC3 cells cultured 72 h after drug administration in the (**A**) control, (**B**) 0.01 μM gemcitabine, (**C**) 0.01 μM gemcitabine + 0.288 μM CG200745, (**D**) 0.01 μM gemcitabine + 0.015 μM erlotinib, (**E**) 0.01 μM gemcitabine + 0.288 μM CG200745 + 0.015 μM erlotinib. Annexin V-FITC/PI double staining; Q1, autophagic cell death; Q2, late apoptotic cells; Q3, normal cells; Q4, early apoptotic cells.

Flow cytometry was performed to examine the apoptotic rate of BxPC3 cells 72 h after CG200745 administration by using the Annexin V-FITC/PI double staining method (Fig. 3). The results of flow cytometry showed that CG200745 treatment increased the number of Annexin V-positive BxPC3 cells, indicating apoptosis induction. Notably, cell exposure to CG200745 resulted in enhanced accumulation of autophagic, late apoptotic cells. Flow cytometry results showed that CG200745 combined with gemcitabine/erlotinib treatment increased the number of Annexin V-positive BxPC cells, indicating apoptosis activation. CG200745 sensitized pancreatic cancer cells to the anti-proliferative effects of gemcitabine and erlotinib. The interaction between CG200745 and gemcitabine/erlotinib was further analyzed using the Chou–Talalay method^15^, to determine whether this combination exhibited additive or synergistic cytotoxicity. Using CompuSyn software, we calculated the Combination index (CI) according to concentrations of CG200745, gemcitabine, and erlotinib, which revealed that this combination was synergistic in the three cell lines. Moreover, the CIs of triple combinations showed better synergism than that of other combinations (Supplementary Table 1).

### Combination effect of CG200745 with gemcitabine/erlotinib in a xenograft model

**Figure 4:**
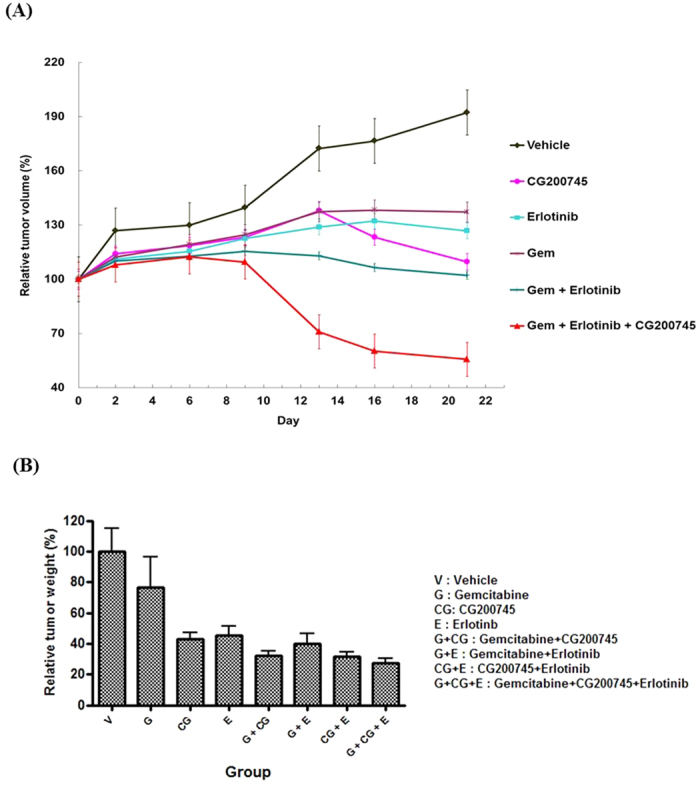
CG200745 inhibits the growth of pancreatic tumors in nude mice. (**A**) Relative tumor volume with gemcitabine, erlotinib, and CG200745 treatments. (**B**) Relative tumor weight with gemcitabine, erlotinib, and CG200745 treatments. Cell line: BxPC-3; animal: Balb/c nude mouse, female, 6 w; dosing schedule: 3 weeks; CG200745: 30 mg/kg, IP, daily; erlotinib: 50 mg/kg, PO, daily; femcitabine, Gem: 20 mg/kg, IP, day 1, 4, 7, 10, 13, 16, 19.

As shown in Fig. 4, the BxPC3 xenograft growth in nude mice was dramatically inhibited by the co-administration of CG200745 and gemcitabine/erlotinib. Combined gemcitabine/erlotinib and CG200745 reduced the tumor volume up to 50% by 3 weeks in the BxPC3 xenograft model.

### Growth inhibition by CG200745 in gemcitabine-resistant pancreatic cancer cells

**Figure 5:**
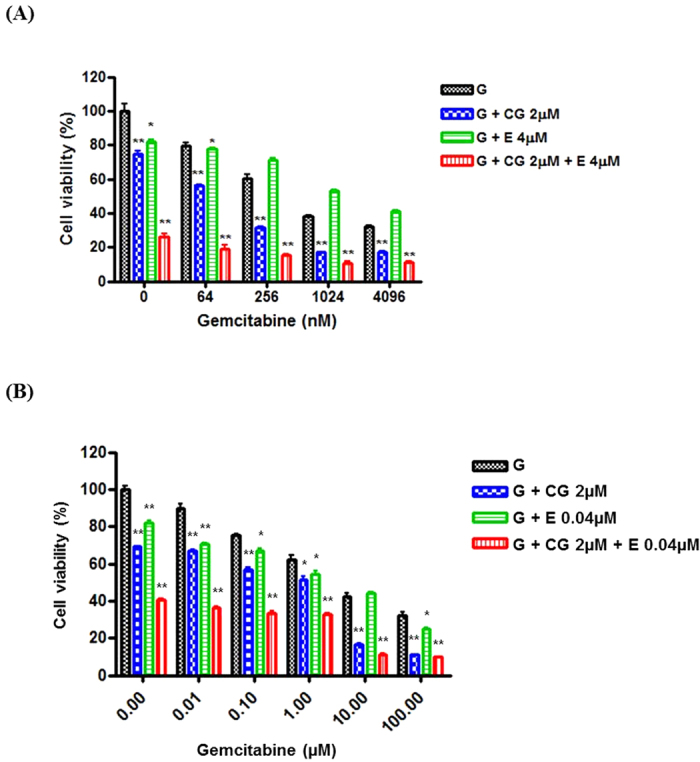
Synergistic effect of CG200745 in combination with gemcitabine/erlotinib in gemcitabine-resistant pancreatic cancer cells (**A**, Cfpac-1; **B**, HPAC). The anti-proliferative effect of CG200745 combined with gemcitabine/erlotinib is more than the effect of gemcitabine/erlotinib without CG200745 in gemcitabine-resistant pancreatic cancer cells. * or **Indicates significant differences compared with control (*p* < 0.05 or *p* < 0.01). G, gemcitabine; CG, CG200745; E, erlotinib.

Gemcitabine-resistant cells were very insensitive to the growth-inhibitory effect of gemcitabine. The IC_50_ of gemcitabine in gemcitabine-resistant cells was 0.595 and 7.654 μM, which was 24.5-fold and 7.9-fold higher than that of parent cell lines Cfpac-1 and HPAC, respectively. However, the treatment with CG200745 in gemcitabine-resistant pancreatic cancer cells eliminated the chemo-resistance (Supplementary Fig. 3). The IC_50_ of CG200745 in gemcitabine-resistant cells was 9.8 and 6.8 μM, which was 0.8-fold and 0.9-fold lower than that of parent cell lines Cfpac-1 and HPAC, respectively. CG200745 sensitized gemcitabine-resistant pancreatic cancer cells to the anti-proliferative effects of gemcitabine and erlotinib. The anti-proliferative effect of the triple combination was most effective in gemcitabine-resistant Cfpac-1- and HPAC-cell lines and CIs revealed that this combination was strongly synergistic (Fig. 5).

**Figure 6:**
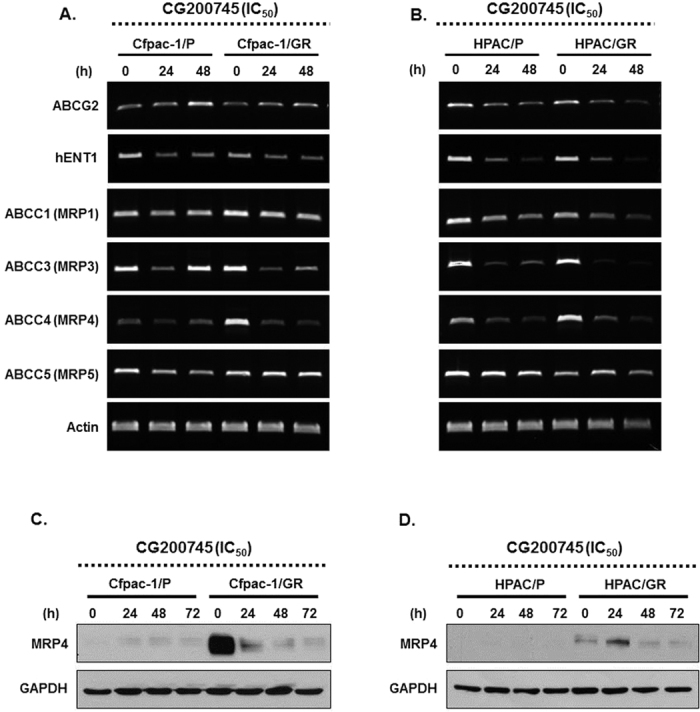
Agarose gel electrophoresis of the MRP family members indicates mRNA expression in gemcitabine-resistant pancreatic cancer cells. CG200745 treatment decreases mRNA levels of ABC transporter genes, especially MRP3 and MRP4 (**A**,**C**: Cfpac-1; **B**,**D**: Hpac). Western blot analysis of MRP4 expression in gemcitabine resistant cells after CG200745 treatment.

The agarose gel electrophoresis of the MRP family revealed that CG200745 treatment decreased the mRNA levels of ABC transporter genes, especially MRP3 and MRP4 (Fig. 6A and B). Western blot analysis showed significantly reduced MRP4 expression in gemcitabine-resistant pancreatic cancer-cell lines (Fig. 6C and D). It was hypothesized that gemcitabine sensitivity could be increased in combination with CG200745. These preclinical results show that combined treatment of CG200745 and gemcitabine/erlotinib potentiated the anti-tumor effects in gemcitabine-resistant pancreatic cancer cells.

## Discussion

Aberrant expression of HDACs represents an attractive therapeutic target for pancreatic cancer. Since HDAC inhibitors can reactivate epigenetically silenced genes, they could be used in pancreatic cancer as anticancer agents. Previously, several HDAC inhibitors have been studied for the inhibition of pancreatic cancer cells. Trichostatin A (TSA) and suberoylanilide hydroxamic acid (SAHA) strongly inhibited the growth of pancreatic cancer cells by inducing cell-cycle arrest and apoptosis^16^. Recently, the synergistic effects of HDAC inhibitors and conventional chemotherapeutic agents have been studied^17^, which showed that a combination of gemcitabine and HDAC inhibitors potently enhanced gemcitabine-mediated growth inhibition and apoptosis in pancreatic cancer cells^9^,^18^,^19^. To the best of our knowledge, this is the first study to show the anticancer effects of a new HDAC inhibitor, CG200745, on pancreatic cancer cells. In the present study, CG200745 combined with conventional chemotherapeutic regimens, gemcitabine/erlotinib, dramatically inhibited pancreatic cancer-cell proliferation *in vitro* and *in vivo*. Furthermore, CG200745 enhanced the sensitivity of gemcitabine-resistant pancreatic cancer cells to gemcitabine treatment.

A limited efficacy in solid tumors and undesirable adverse reactions of HDACIs were reported in previous clinical studies^20^. For example, Richards *et al*.^21^ showed the anticancer effect of the combination of the HDAC inhibitor, CI-994, and gemcitabine in pancreatic cancers; patients receiving CI-994 combined with gemcitabine had a higher incidence of grade 3/4 adverse events such as thrombocytopenia, anemia, and leukopenia than those treated with only gemcitabine. However, CG200745 was well tolerated at the tested doses with no dose-limiting toxicities in the first human study. Only grade 3/4 hematologic toxicities were reported, such as anemia and neutropenia, which did not last more than one week. Other toxicities included Grade 1/2 mild fatigue and anorexia^13^.

Gemcitabine-based regimens remain a major chemotherapy for pancreatic cancer, but the resistance to gemcitabine has negatively affected the overall survival in cancer patients. Drug resistance is attributable to several processes occurring in neoplastic cells. One of these processes is decreased drug accumulation within cancer cells because of an increased drug efflux. Specifically, the ATP-binding cassette transporter family C (ABCC) is responsible for mediating multidrug resistance^22^. Human ABCC consists of 12 members, 9 of which comprise a group of multidrug-resistant proteins (MRP1–MRP9, ABCC1–ABCC6, and ABCC10–ABCC12). In the present study, CG200745 decreased the mRNA transcripts and its corresponding protein for MRP4 *in vitro*. Therefore, CG200745 controlled the drug efflux and sensitivity in gemcitabine-resistant pancreatic cancer cells. Improvement of resistance to gemcitabine allows this drug to be used more effectively to treat pancreatic cancer.

The HDAC family is a multiclass consisting of 18 HDACs divided into four subgroups by cell localization, role, and structure: class I including HDAC 1, 2, 3, and 8 (localized in the nucleus); class II HDACs including 4, 5, 6, 7, 9, and 10 (localized in both the nucleus and cytoplasm); class III HDACs consists of sirtuins (1–7); and class IV includes HDAC 11^23^. Similar to the overexpression of HDAC1 in gastric cancer, and HDAC2 and HDAC3 in colon cancer, HDAC7 is over-expressed in pancreatic cancer^8^,^24^. HDAC7 is also an important regulator in cancer^25^. Approximately 81% of the PDAC tissue samples displayed increased HDAC7 mRNA and protein expressions. Therefore, previous studies showed that HDAC7 could be critical in tumor growth and metastasis, and a clinically beneficial biomarker for pancreatic cancer diagnosis and prognosis^8^,^24^. In the present study, high levels of HDAC7 mRNA and protein were detected in pancreatic cancer cells (Supplementary Fig. 4). The inhibitory effect of our novel compound on HDAC7 in pancreatic cancer cells was considerable.

In Cfpac cell, histone acetylation decreased in 48 and 72 h gradually with decreased expression of p21 and BAX than two other cell lines. When we re-tested the Western Blot for histone acetylation in Cfpac-1 cell, the expression of histone acetylation was decreased like the previous result. But, the pattern of p21 and BAX expression was similar with other cell lines (Supplementary Fig. 5). The difference of histone acetylation expression depends on the different characteristics of pancreatic cancer cell lines. The difference of genetic background for 3 pancreatic cancer cell lines used in this study may be one of the reasons^26^. In a previous study, Western Blot analysis showed different expression pattern of histone acetylation in 2 patients. In the case of patient 01, histone acetylation decreased in 24 and 48 h, but the expression of histone acetylation increased in 24 and 48 h in another patient. According to pancreatic cancer cell lines, the extent or timing of histone acetylation expression was different^27^. In the present study, Cfpac-1 cell lines seem to respond for histone acetylation rapidly compared to other cell lines.

In conclusion, the novel HDAC inhibitor, CG200745, showed an antitumor effect in three types of pancreatic cancer cell lines and synergized the antitumor effect of gemcitabine and erlotinib, which are conventional chemotherapeutic regimens for pancreatic cancer cells. We also showed that the CG200745 combined treatment induced cell death in gemcitabine-resistant cells, suggesting that CG200745 can overcome gemcitabine resistance. Resolving the problem of drug resistance to conventional therapies is a key factor for improving pancreatic cancer prognosis. Our data provided evidence that CG200745 could potentially improve the treatment of gemcitabine-resistant pancreatic cancer. CG200745 combined with standard gemcitabine-based chemotherapy could be used as a successful combination therapy in the future.

## Methods

### Chemicals

CG200745 was provided by CrystalGenomics Inc. (Seoul, Rep. Korea)^11^,^12^. A 50-mM stock solution for biological assays was prepared in dimethyl-sulfoxide (DMSO) and stored at −20 °C until use. Erlotinib was purchased from ApexBio (ApexBio Technology, Houston, TX, USA), and gemcitabine was supplied by Eli Lilly Korea (Seoul, Korea).

### Cell lines

Three pancreatic cancer-cell lines, BxPC3, Cfpac-1, and HPAC, were purchased from the American Type Culture Collection (ATCC, Manassas, VA, USA). BxPC-3 cells were grown in RPMI1640 (Invitrogen Gibco, Grand Island, NY, USA) with 10% fetal bovine serum (FBS; Hyclone, Logan, Utah, USA), CFPAC-1 cells were grown in IMDM (Invitrogen Gibco) with 10% FBS (Hyclone), and HPAC cells were grown in DMEM/F12 (Invitrogen Gibco) with 10% FBS (Hyclone). Cells were maintained in a humidified incubator with 5% CO_2_ at 37 °C.

### Cell viability assay

Cells were seeded at 3~5 × 10^3^/well in 96 well plates and exposed to various concentrations of compounds for 72 h. Cell viability effects of CG200745, gemcitabine, and erlotinib were assessed by the 3-(4,5-dimethylthiazol-2yl)-2,5-diphenyltetrazolium bromide (MTT; Amresco, Solon, OH, USA) assay. IC_50_ values were reached when cell growth was inhibited at 50% of the DMSO control. Values are the means of triplicate wells from three independent experiments for each drug concentration.

### Western blotting

Cells were lysed in a lysis buffer containing 70 mM glycerophosphate (pH 7.2), 0.6 mM Na vanadate, 2 mM MgCl_2_, 1 mM EGTA, 1 mM DTT, 0.5% Triton X-100, 0.2 mM phenylmethylsulfonyl fluoride (PMSF), and 1× complete protease inhibitor (Roche Applied Science, Nutley, NJ, USA). Protein (25 μg) was resolved on SDS-polyacrylamide gels and transferred to polyvinylidene difluoride (PVDF) membranes (Immobilon-P, Millipore, Bedford, MA, USA). The membranes were blocked in 5% (w/v) nonfat dry milk (Bio-Rad Laboratories, Inc., Hercules, CA, USA) and incubated with the following primary antibodies: mouse monoclonal anti-HDAC1, mouse monoclonal anti-HDAC2, mouse monoclonal anti-HDAC3, rabbit monoclonal anti-HDAC4, rabbit monoclonal anti-HDAC6, rabbit polyclonal anti-phospho-AKT, rabbit polyclonal anti-phospho-ERK (Cell Signaling Technology, Inc., Danvers, MA, USA), mouse monoclonal anti-HDAC7, rabbit polyclonal anti-EGFR, rabbit polyclonal anti-acetylated histone h3, rabbit polyclonal anti-cmet, mouse monoclonal anti-caspase3, rabbit polyclonal anti-p21, rabbit polyclonal anti-BAX, rabbit polyclonal anti-AKT, rabbit polyclonal anti-ERK, mouse monoclonal anti-MRP4, and mouse monoclonal anti-GAPDH (Santa Cruz Biotechnology, Santa Cruz, CA, USA). After incubation with appropriate horseradish peroxidase-conjugated secondary antibodies (Santa Cruz Biotechnology), immunoblots were developed with the West Pico Chemiluminescent substrate (Thermo Scientific, Rockford, IL, USA).

### Semi-quantitative RT-PCR

Total RNA was extracted using an RNeasy mini kit (QIAGEN, Hilden, Germany), and cDNA was synthesized using a Superscript II system (Invitrogen) according to manufacturer’s protocols. Beta-actin (*ACTB*) was used as a reference gene. The primers are listed in Supplementary Table 2.

### Immunofluorescence

Cells were grown on coverslips and treated with indicated concentrations of compounds for 48 h. Cells were fixed with 4% paraformaldehyde and incubated with blocking buffer (1 × PBS/5% normal donkey serum/0.3% Triton X-100). Cells were stained with rabbit polyclonal anti-cleaved caspase-3 (1:400, Cell Signaling Technology, Inc.) overnight at 4 °C. Stained cells were incubated with goat anti-rabbit Alexa 488-conjugated secondary antibody (Jackson ImmunoResearch Inc., West Grove, PA, USA) for 1 h. Nuclei were labeled with 4′,6-diamidino-2-phenylindole (DAPI). The stained cells were analyzed on an Olympus BX51 microscope and images were captured using an Olympus DP71 camera (Olympus America Inc., Center Valley, PA, USA).

### Apoptosis

Apoptosis was detected by flow cytometry through Annexin V-FITC/propidium iodide (PI) staining. Cells (2 × 10^5^ cells per well) were seeded on 6 well plates plates and incubated overnight at 37 °C. The compound was added and cells were incubated for a further 72 h. Trypsinized cells were harvested, with 100 μl 1× binding buffer. Harvested cells were incubated with 1 μg/ml FITC-labeled Annexin V (BD Biosciences, San Diego, CA) and 2.5 μg/ml PI at room temperature of 25 °C for 15 min in the dark. Flow cytometric measurement of the stained cells was performed with BD LSRII (BD Bioscience, San Jose, CA).

### Xenograft mouse model

Experiments carried out using 6-week-old male BALB/c nude mice (Japan SLC, Inc., Japan) were approved by the Institutional Animal Care and Use Committee (IACUC) of Biotoxtech Co., Ltd. based on the Animal Protection Act. All experiments were performed in accordance with relevant guidelines and regulations. Exponentially grown BxPC3 cells (5 × 10^6^ cells/mouse) were s.c. injected into the right flank. Treatment started two weeks after the tumor implant, when tumors reached a volume of approximately 100 mm^3^. Gemcitabine (20 mg/kg) was administered i.p. for three weeks (day 1, 4, 7, 10, 13, 16, 19). Erlotinib (50 mg/kg) was administered p.o. daily for three weeks. CG200745 (30 mg/kg) was administered i.p. daily for 3 weeks. Tumor formation was monitored twice a week by measuring the width and length of the mass, and tumor volume (TV) was calculated using the formula ‘TV (mm^3^) = (L × W^2^)/2’, with L as the largest and W as the smallest diameters. Animals were sacrificed after three weeks from the first administration.

### Gemcitabine-resistant pancreatic cancer-cell lines

Gemcitabine-resistant pancreatic cancer cells were established by escalating doses of gemcitabine serially in HPAC and CFPAC-1 cells^28^. Initially, cells were cultured for 72 h with IC_50_ of gemcitabine with a defined drug-free interval. As cells adapted to the dose, the gemcitabine concentration was serially doubled. Finally, after cells recovered from 10 μM gemcitabine treatment, 100 μM of the drug was added to the medium to eradicate most of the cell population.

### Combination studies

The effectiveness of chemotherapeutic agents used in this study in combination, was analyzed using CompuSyn software^15^,^29^. CI values were calculated to confirm synergy. CI < 0.9 indicates synergistic effects, CI between 0.9 and 1.1 indicates addictive effects, and CI > 1.1 indicates antagonistic effects.

### Statistics

Statistical analyses were performed using SPSS 18.0 software. All values were expressed as the mean ± standard deviation. Comparisons between two groups were analyzed using t-tests. Values of *P* < 0.05 were considered significant.

## Additional Information

**How to cite this article:** Lee, H. S. *et al*. A novel HDAC inhibitor, CG200745, inhibits pancreatic cancer cell growth and overcomes gemcitabine resistance. *Sci. Rep.* **7**, 41615; doi: 10.1038/srep41615 (2017).

**Publisher's note:** Springer Nature remains neutral with regard to jurisdictional claims in published maps and institutional affiliations.

## Supplementary Material

Supplementary Information
