# Supplementary material for: A novel HDAC inhibitor, CG200745, inhibits pancreatic cancer cell growth and overcomes gemcitabine resistance
Source: Sci Rep. 2017 Jan 30;7:41615. doi: 10.1038/srep41615 (PMC5278546; doi:10.1038/srep41615)
Supplement: Supplementary Information [file srep41615-s1.pdf]

A novel HDAC inhibitor, CG200745, inhibits pancreatic cancer cell growth and overcomes gemcitabine resistance

Hee Seung Lee[1], Soo Been Park[1], Sun A Kim[1], Sool Ki Kwon[2], Hyunju Cha[2], Do Young Lee[2], Seonggu Ro[2], Joong Myung Cho[2], Si Young Song[1†]

[1]*Division of Gastroenterology, Department of Internal Medicine,*

Yonsei University College of Medicine, Seoul, Korea

[2]*CrystalGenomics, Inc., 5F, Bldg A, Korea Bio Park, Seongnam, Korea*

Running Title: HDAC inhibitor, CG200745, in pancreatic cancer

Corresponding author:

Si Young Song, MD, PhD

Division of Gastroenterology, Department of Internal Medicine,

Yonsei University College of Medicine,

50-1 Yonsei-ro, Seodaemun-gu, Seoul 120-752, Korea

Phone: +82-2-2228-1957 Fax: +82-2-2227-7900

E-mail: sysong@yuhs.ac

Supplementary Figure 1. Anti-proliferative activities of erlotinib against three pancreatic cancer cell lines and two gemcitabine-resistant cell lines. Cell viability curve based on the erlotinib concentration in five pancreatic cancer cell lines. The doses of erlotinib equivalent to IC 20~30 were selected.

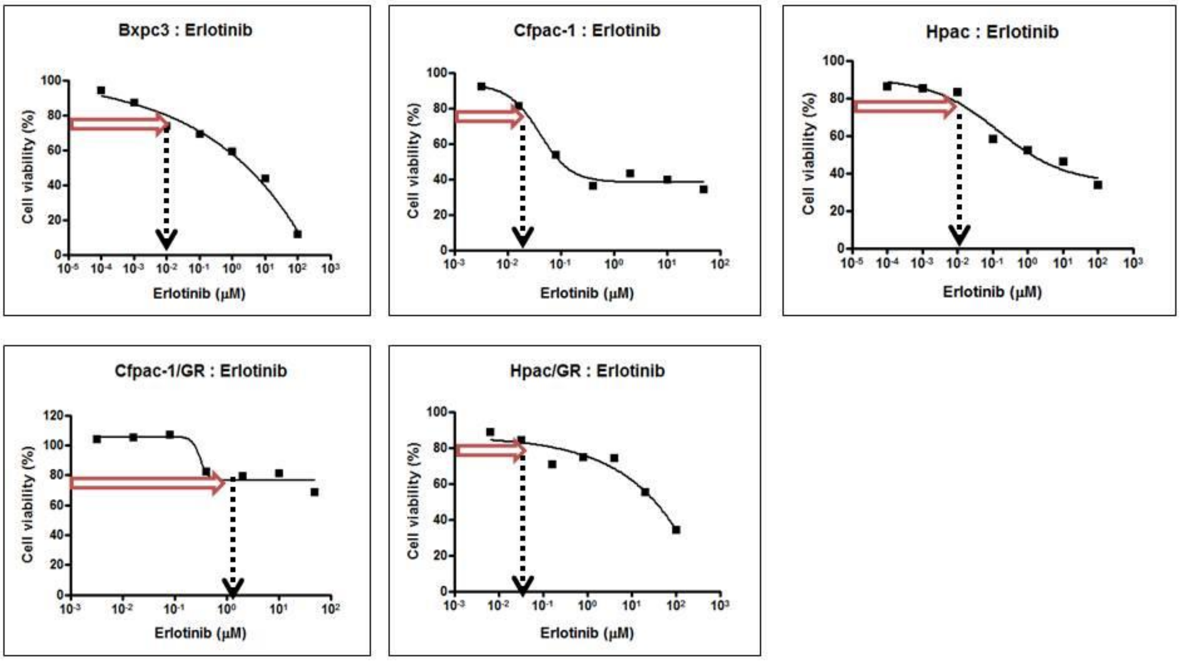

Supplementary Figure 2. Synergistic effect of CG200745 combined with gemcitabine/erlotinib (A, Cfpac1; B, HPAC). The doses of erlotinib and CG200745 were equivalent to IC 20~30. The growth of pancreatic cells was analyzed via an MTT assay after treatment with various concentrations of gemcitabine over a time-course (0–72 h). The anti-proliferative effect of CG200745 with gemcitabine/erlotinib is more enhanced than the effect of gemcitabine/erlotinib without CG200745 in pancreatic cancer cells. Western Blot analysis to investigate the pancreatic cancer cell apoptosis and analyze the molecular pathway related to CG200754. CG200745 combined with gemcitabine/erlotinib induces apoptosis through caspase-3 activation. Immunofluorescent staining of cleaved caspase-3 expressing cells. Fluorescence signals specific to cleaved caspase-3 antibodies were visualized as green, and DAPI (blue) was used to indicate nuclei. * or ** indicates significant differences compared with the control ($p < 0.05$ or $p < 0.01$). G, gemcitabine; CG, CG200745; E, erlotinib

(A)

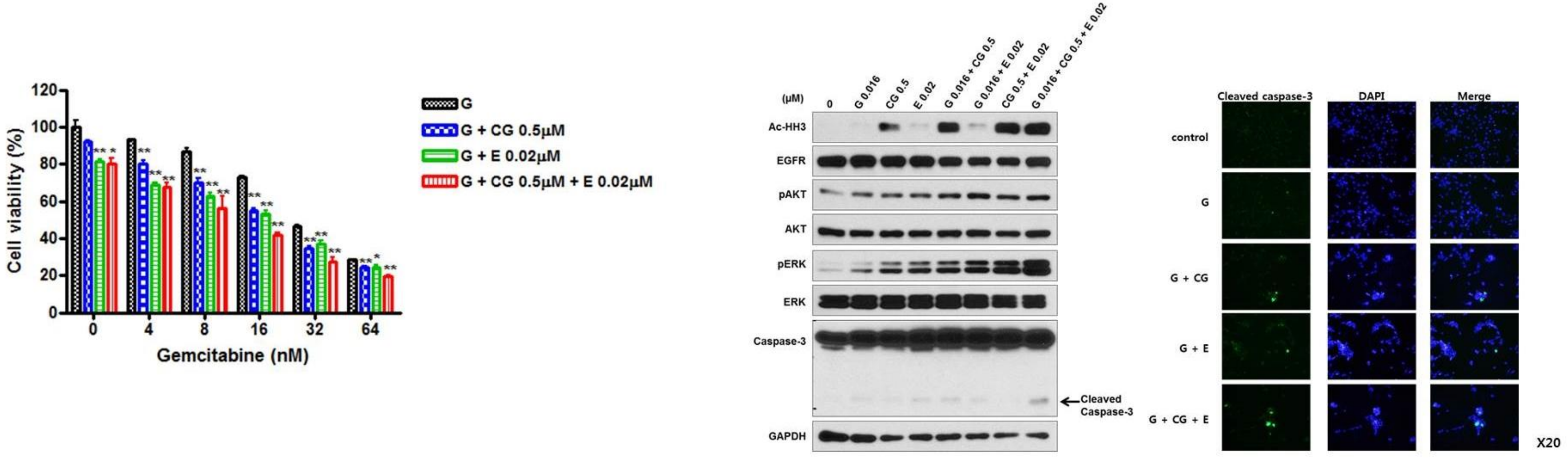
Cell viability (%)
Gemcitabine (nM)
0
4
8
16
32
64
G
G + CG 0.5μM
G + E 0.02μM
G + CG 0.5μM + E 0.02μM
(μM)
0
G 0.016
CG 0.5
E 0.02
G 0.016 + CG 0.5
G 0.016 + E 0.02
CG 0.5 + E 0.02
G 0.016 + CG 0.5 + E 0.02
Ac-HH3
EGFR
pAKT
AKT
pERK
ERK
Caspase-3
Cleaved Caspase-3
GAPDH
Cleaved caspase-3
DAPI
Merge
control
G
G + CG
G + E
G + CG + E
X20

(B)

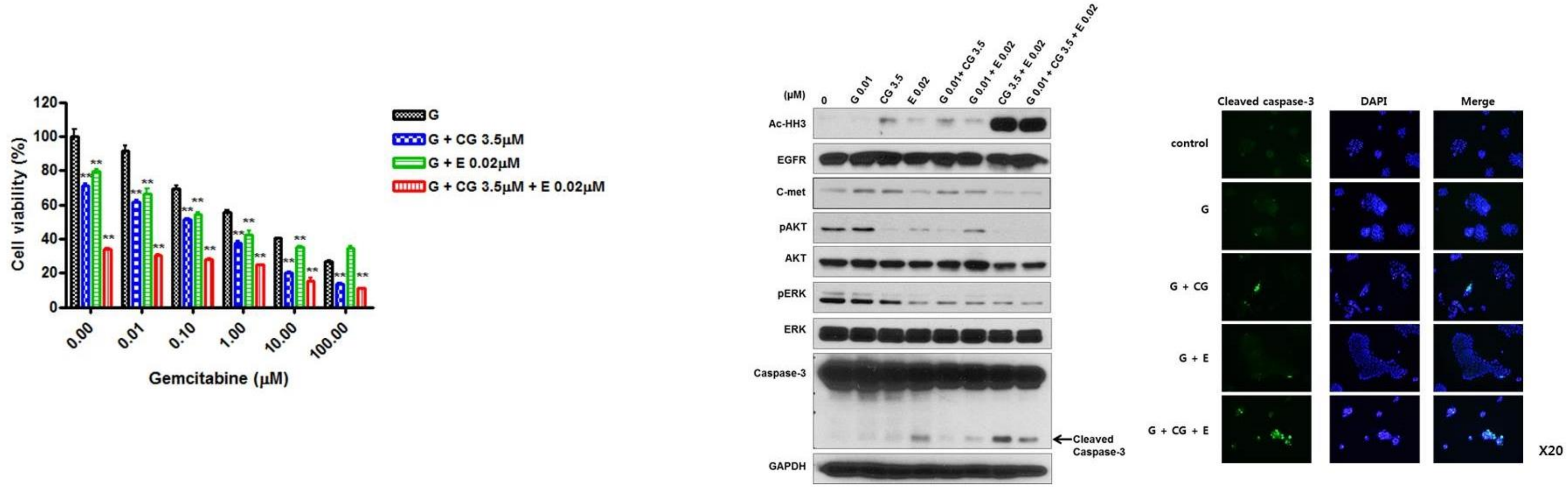
Cell viability (%)
Gemcitabine (μM)
0.00
0.01
0.10
1.00
10.00
100.00
G
G + CG 3.5μM
G + E 0.02μM
G + CG 3.5μM + E 0.02μM
(μM)
0
G 0.01
CG 3.5
E 0.02
G 0.01+ CG 3.5
G 0.01 + E 0.02
CG 3.5 + E 0.02
G 0.01 + CG 3.5 + E 0.02
Ac-HH3
EGFR
C-met
pAKT
AKT
pERK
ERK
Caspase-3
Cleaved Caspase-3
GAPDH
Cleaved caspase-3
DAPI
Merge
control
G
G + CG
G + E
G + CG + E
X20

Supplementary Figure 3. Cell viability curve based on the concentration of CG200745 and gemcitabine in gemcitabine-resistant cell lines (A, gemcitabine-resistant Cfpac-1; B, gemcitabine-resistant HPAC)

(A)

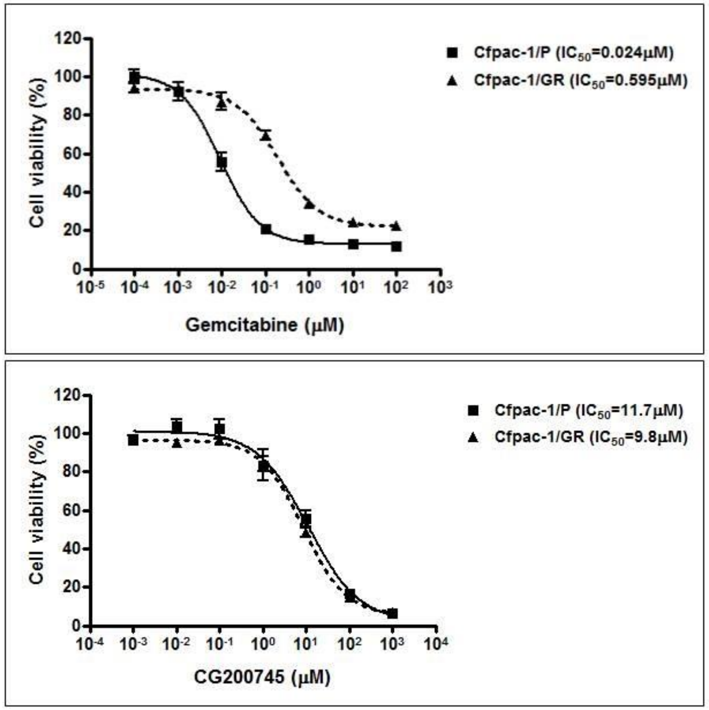

(B)

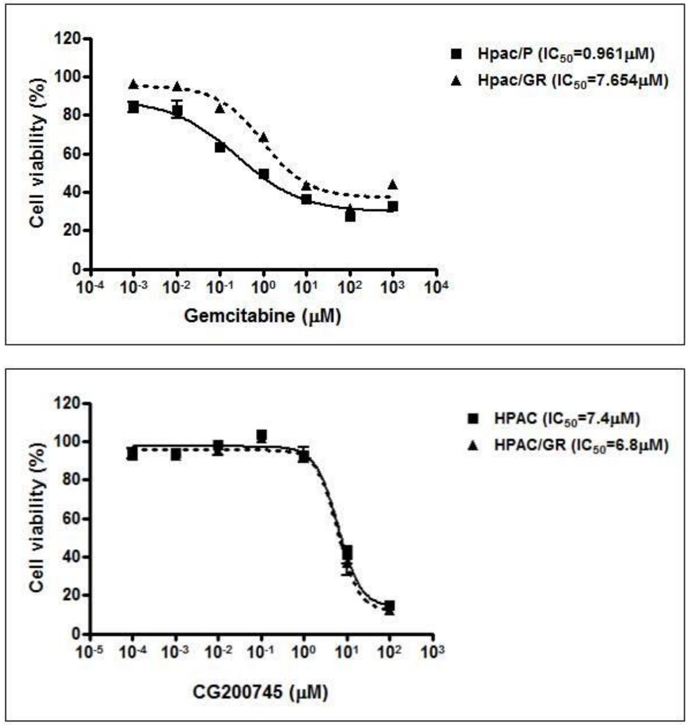

Supplementary Figure 4. HDAC inhibitor, CG200745 selectively decreases HDAC7 expression in gemcitabine-resistant pancreatic cancer cells.

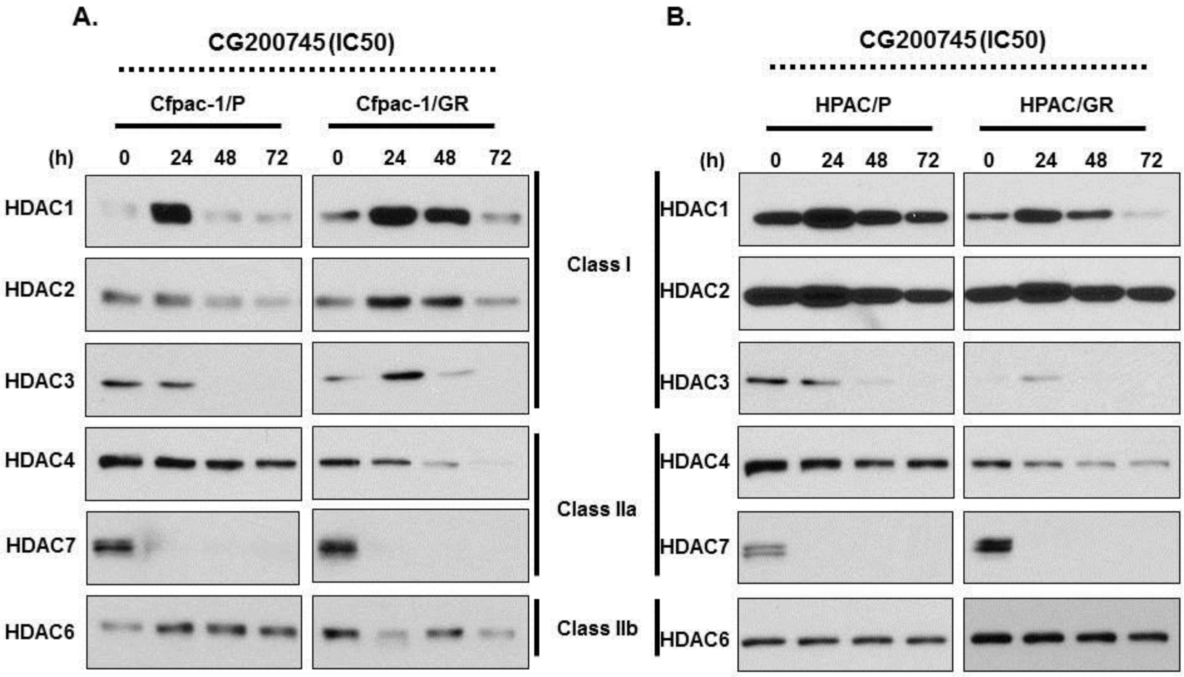

Supplementary Figure 5. Anti-proliferative and pro-apoptotic activities of CG200745 against pancreatic cancer cells. CG200745 induces histone-H3 acetylation and increases BAX and p21 expression related to apoptosis, Cell line; Cfpac-1.

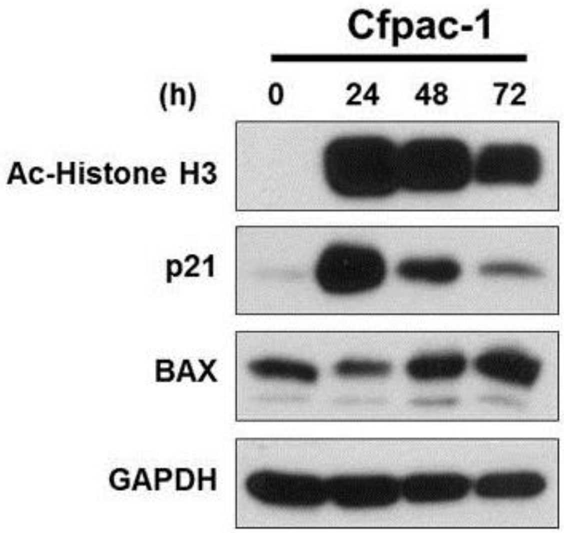

Supplementary table 1. Combination index (CI) according to concentration of CG200745, gemcitabine, and erlotinib in the three pancreatic cancer cell lines and two gemcitabine-resistant cell lines (A, BxPC3; B, Cfpac1; C, HPAC; D, gemcitabine-resistant Cfpac-1; E, gemcitabine-resistant HPAC). CI < 0.1, very strong synergism; CI 0.1–0.3, strong synergism; CI 0.3–0.9, synergism; CI 0.9–1.1, addictive effect; CI > 1.1, antagonism

(A)

Combination index	0.74	0.70	0.47	0.45
Gemcitabine(μM)	0.001	0.010	0.100	1.000
Erlotinib(μM)	0.015	0.015	0.015	0.015
CG200745(μM)	0.288	0.288	0.288	0.288

(B)

Combination index	0.73	0.59	0.54	0.53	0.64
Gemcitabine(nM)	4	8	16	32	64
Erlotinib(μM)	0.02	0.02	0.02	0.02	0.02
CG200745(μM)	0.5	0.5	0.5	0.5	0.5

(C)

Combination index	0.52	0.51	0.49	0.41	0.41
Gemcitabine(μM)	0.01	0.10	1.00	10.00	100.00
Erlotinib(μM)	0.02	0.02	0.02	0.02	0.02
CG200745(μM)	3.5	3.5	3.5	3.5	3.5

(D)

Combination index	<0.01	<0.01	<0.01	0.01
Gemcitabine(nM)	64	256	1024	4096
Erlotinib(μM)	4	4	4	4
CG200745(μM)	2	2	2	2

(E)

Combination index	0.42	0.38	0.38	0.14	0.13
Gemcitabine(μM)	0.01	0.10	1.00	10.00	100.00
Erlotinib(μM)	0.04	0.04	0.04	0.04	0.04
CG200745(μM)	2	2	2	2	2

Supplementary Table 2. Primer sequences used for RT-PCR

**Gene**	**Sense**	**Antisense**
ABCG2	TATGAGTGGCTTATCCTGCT	CACTGATCCTTCCATCTTGT
hENT1	GACAACCAGTCACCAGCCTCAG	AGAGCATCCAGCTGCACCTTCA
MRP1	CTGACAAGCTAGACCATGAATGT	TCACACCAAGCCGGCGTCTTT
MRP3	GGACCCTGCGCATGAACCTG	AGGCAAGTCCAGCATCTCTGG
MRP4	GGATCCAAGAACTGATGAGTTAAT	TCACAGTGCTGTCTCGAAAATAG
MRP5	GCTGTTCAGTGGCACTGTCAG	TCAGCCCTTGACAGCGACCTT
β–Actin	GGCATCCTCACCCTGAAGTA	GGGGTGTTGAAGGTCTCAAA
